# Supplementary material for: WordCommentsAnalyzer: A windows software tool for qualitative research
Source: F1000Res. 2018 Sep 4;7:536. Originally published 2018 May 3. [Version 2] doi: 10.12688/f1000research.14819.2 (PMC6137411; doi:10.12688/f1000research.14819.2)
Supplement: Supplementary file 3 [file f1000research-7-17614-s0002.tgz › b2a79496-4ada-47c4-9682-a99ce867836d.zip!/Eight Iranian Health Journals/Salmand_Iranian Journal of Ageing _24.docx]

© Salmand_Iranian Journal of Ageing . This work is licensed under CC BY-NC 4.0 (https://creativecommons.org/licenses/by-nc/4.0/).

Volume & Issue: دوره 7 - شماره 2

Title: مناسب‌سازی منظر شهری برای سالمندان

http://salmandj.uswr.ac.ir/article-1-622-fa.html

اهداف: رشد و گسترش شهرها و تغییرات به وجود آمده در طرح‌ها و مناظر شهری همگام با افزایش جمعیت و سیر صعودی سن افراد هماهنگی لازم را نداشته و از ارایه و انجام بسیاری از طرح‌ها به خوبی می توان دریافت که توجه لازم به همه اقشار جامعه از جمله سالمندان و معلولین نمی شود. بدین ترتیب همواره قشرهایی از مردم هستند که با مشکلات عدیده اعم از مناسب سازی محیط و عدم وجود منظر مناسب شهری روبرو بوده و جایگاه مناسبی برای حضور نمی یابند.  مواد و روش‌ها: سالمندان از جمله اقشاری هستند که با مشکلات زیادی در شهر مواجه می‌باشند. با وجود تمایل سالمندان برای شرکت در تعاملات اجتماعی و استفاده از فضای شهری، این دسته از افراد از فضای شهری مناسبی که امکان زندگی مستقل را برای آنان فراهم سازد، برخوردار نیستند. به بیان دیگر، سالمندان دوست دارند تا با انجام مسئولیت ها و ایجاد روابط اجتماعی با همسایگان، ثابت کنند که همچنان از توانایی دوران جوانی برخوردار هستند. اما آیا فضاهای شهری در شهرهای ما نیازهای سالمندان را برآورده می‌کنند و آیا تسهیلات مناسب را برای این قشر از جامعه تأمین می‌نمایند؟ یافته‌ها: به یقین عوامل متعددی نظیر کاربری اراضی، بافت شهری، دسترسی و منظر شهری در طراحی شهر تاثیرگذار هستند. این پژوهش به بررسی تأثیر عامل منظر شهری بر تعامل اجتماعی سالمندان در شهر می‌پردازد. نتیجه‌گیری: بدیهی است که این عامل نقش بسیار مهمی در مسیر یابی داشته و با نیازهای سالمندان و اختلالات بینایی و شناختی آنان مرتبط می باشد. مقاله حاضر به بررسی این مسئله در محله قیطریه تهران (به عنوان نمونه موردی) می‌پردازد و در پایان راهنمای مرتبط با این مسئله را ارائه می‌دهد.

Title: بررسی و مقایسه شادکامی و حرمت خود در سالمندان مقیم مراکز شبانه‌روزی و سالمندان مقیم در خانه

http://salmandj.uswr.ac.ir/article-1-621-fa.html

اهداف: در این پژوهش، رابطه حرمت خود و شادکامی و مقایسه این دو متغیر در دو گروه سالمندان مقیم مراکز شبانه روزی و مقیم‌ خانه مورد بررسی قرار گرفت. مواد و روش‌ها: مطالعه حاضر توصیفی و از نوع همبستگی است در این پژوهش برای سالمندان مقیم در مراکز شبانه روزی و سالمندان مقیم در خانه از نمونه گیری دردسترس استفاده می شود. تعداد افراد جامعه درگروه مراکز شبانه روزی416 نفر بودند که طبق جدول مورگان 60 سالمند مقیم در مراکز شبانه روزی (30 زن و 30 مرد) به عنوان نمونه پژوهش و برای مقایسه 60 نفر از سالمندان مقیم در خانه (30 زن و 30 مرد) به عنوان نمونه دیگر پژوهش تعیین شدند، که جمعاً 120 نفر می‌شوند. برای جمع آوری اطلاعات از پرسشنامه جمعیت‌شناختی، پرسشنامه شادکامی آکسفورد و پرسشنامه حرمت خود استفاده شد. به منظور تجزیه و تحلیل اطلاعات از همبستگی پیرسون و آزمون t برای دو گروه مستقل استفاده شد. یافته‌ها: رابطه حرمت خود و شادکامی در دو گروه از لحاظ آماری در سطح کمتر از 0/01 معنادار بود و تفاوت میانگین حرمت خود و شادکامی سالمندان مقیم مراکز شبانه روزی با سالمندان مقیم در خانه اختلاف آماری معناداری نشان دادند(0/01>P). نتیجه‌گیری: سالمندان مقیم خانه، دارای حرمت خود و شادکامی مطلوب‌تری نسبت به سالمندان مقیم مراکز شبانه روزی بودند.

Title: بررسی کیفیت رژیم غذایی با استفاده از شاخص تغذیه سالم 2005 در سالمندان مناطق شهری استان مرکزی در سال 1389

http://salmandj.uswr.ac.ir/article-1-623-fa.html

اهداف: جمعیت سالمندان در کشورمان به سرعت در حال افزایش است و کیفیت تغذیه به عنوان یک عامل موثر بر وضعیت سلامتی این گروه سنی کمتر مورد بررسی قرار گرفته شده است. هدف از این مطالعه ارزیابی کیفیت رژیم غذایی سالمندان 60 ساله و بالاتر ساکن مناطق شهری استان مرکزی با استفاده از شاخص تغذیه سالم 2005 و ارتباط آن با متغیرهای جمعیتی و اجتماعی‌اقتصادی بود. مواد و روش‌ها: این پژوهش از نوع توصیفی است که بر روی 165 سالمند در مناطق شهری استان مرکزی انجام شده است. روش نمونه گیری به‌صورت تصادفی دو مرحله‌ای بود، برای جمع آوری اطلاعات از پرسشنامه عمومی و سه یادآمد غذایی 24 ساعته استفاده شد، سپس کیفیت رژیم غذایی افراد بر اساس شاخص تغذیه سالم 2005 که یک شاخص 100 امتیازی روا و پایا است، تعیین شد. یافته‌ها: میانگین کلی شاخص تغذیه سالم 2005 برابر 54/08درصد بود. درصد افرادی که گروه‌های غذایی را در حد مطلوب مصرف کرده بودند به این شرح بود: غلات 93/3، غلات کامل صفر، میوه ها 70/9، میوه کامل 84/2، سبزیجات 47/9، سبزیجات تیره-نارنجی-حبوبات 12/7، لبنیات 37، گوشت‌ها 40، روغن‌های گیاهی 18/8، چربی‌های اشباع 40، سدیم 1/8 و کالری‌های اضافی ناشی از چربی‌های جامد و شکر 1/2 درصد. همچنین امتیاز کلی شاخص فوق با بالا رفتن سطح سواد (P<0/01)، درآمد و مدت تماشای تلویزیون (P<0/05) افزایش و با بالاتر رفتن سن (P<0/001) کاهش یافت. نتیجه‌گیری: با توجه به پایین بودن کیفیت رژیم غذایی سالمندان مناطق شهری استان مرکزی، احتمالاً همه سالمندان این مناطق از بهبود رژیم غذایی خود سود خواهند برد. آنان نیاز دارند که دریافت غلات کامل، سبزیجات تیره و نارنجی و حبوبات و روغن‌های گیاهی را در الگوی غذایی خود افزایش و دریافت سدیم و به‌خصوص دریافت کالری‌های اضافی ناشی از چربی‌های جامد و شکر را کاهش دهند. همچنین برنامه‌ریزی مسئولین جهت افزایش سطح سواد، درآمد و دانش تغذیه‌ای می‌تواند در رسیدن به این اهداف موثر باشد.

Title: تأثیر هشت هفته تمرین مقاومتی بر هورمون استروژن و درصد چربی بدن در زنان یائسه غیرفعال

http://salmandj.uswr.ac.ir/article-1-624-fa.html

اهداف: هدف از انجام این پژوهش بررسی تأثیر هشت هفته تمرین مقاومتی بر سطح پلاسمایی استروژن در زنان یائسه غیرفعال بود. مواد و روش‌ها: 27 نفر زن یائسه سالم غیرفعال با میانگین سن 7/26±62/59 سال و وزن 7/81±64/96 کیلو گرم به صورت هدفمند برای شرکت در این پژوهش نیمه تجربی انتخاب و سپس به صورت تصادفی به دو گروه تجربی (13 نفر) و شاهد (14 نفر) تقسیم شدند. نمونه های ناشتایی خون ۲۴ ساعت قبل و ۴۸ ساعت بعد از برنامه ی تمرین مقاومتی گرفته شد. شدت تمرین مقاومتی 60 تا 80درصد یک تکرار بیشینه بود و 2 جلسه در هفته به مدت 8 هفته برگزار گردید. برای بررسی تفاوت های درون‌گروهی از t همبسته و برای تعیین تفاوت‌های بین گروهی از t مستقل استفاده شد. یافته‌ها: مقایسه دو گروه کنترل و تجربی نشان داد که سطح استروژن پلاسما پس از 8 هفته تمرین مقاومتی به‌طور معنی‌داری افزایش (0/003=P) و ضربان قلب استراحتی (0/010=P) به طور معنی داری کاهش یافت. درصد چربی آزمودنی ها در مقایسه ی درون گروهی داده های پیش آزمون با پس آزمون در گروه تجربی به طور معنی داری افزایش یافت (0/002=P). نتیجه‌گیری: تمرین مقاومتی ممکن است سطح پلاسمایی هورمون استروژن زنان یائسه را افزایش داده و آنان را از مصرف این هورمون بی نیاز ساخته یا مقدار مورد نیاز آن ها را کاهش دهد. افزایش سطح این هورمون را می توان به افزایش تولید این هورمون در بافت چربی و سایر بافت های پیرامونی نسبت داد.

Title: اثر اختلالات دستگاه شناختی و دستگاه شنوایی مرکزی بر میزان بهره‌مندی از وسایل تقویت‌کننده شنوایی در سالمندان

http://salmandj.uswr.ac.ir/article-1-625-fa.html

اهداف: بسیاری از سالمندان اغلب دارای اختلالاتی در هنگام دنبال کردن گفتار سریع و یا درک گفتار در محیط های پر سرو صدا هستند. این اختلالات احتمالا" ناشی از: 1. آسیب دستگاه شنوایی محیطی؛ 2. تغییرات ساختاری در دستگاه شنوایی مرکزی؛ 3. تغییر در عملکردهای شناختی می باشد. معمول ترین اقدام برای رفع مشکلات شنوایی در سالمندان تجویز وسایل تقویت کننده (سمعک) می‌باشد. امروزه با وجود کارایی بالای وسایل تقویت‌کننده، همچنان گزارشات متعددی از نارضایتی سالمندان مطرح می‌باشد. هدف از این مطالعه بررسی اختلالات شناختی و پردازش شنوایی مرکزی به ویژه در سالمندانی است که استفاده مناسبی از وسایل تقویت‌کننده خود نمی‌برند. مواد و روش‌ها: 19 فرد سالمند به صورت غیر تصادفی در این مطالعه شرکت داشتند. برای همه افراد پرسشنامه استاندارد شده MMSE جهت بررسی وضعیت شناختی بکار گرفته شد. همچنین آزمون اعداد دایکوتیک (DDTs) برای همه سالمندان اجرا گردید. میزان کاهش شنوایی محیطی و میزان رضایتمندی از وسیله تقویت‌کننده، با استفاده از پرسشنامۀ «سنجش رضایتمندی از سمعک در زندگی روزمره» مورد ارزیابی قرار گرفت. نتایج حاصل از آزمون‌ها و پرسشنامه‌های فوق توسط آزمون کروسکال والیس تجزیه و تحلیل گردید. یافته‌ها: بعد از تطبیق سن و میزان افت شنوایی، همبستگی معناداری بین میزان اختلالات شناختی (MMSE) و میزان رضایتمندی از سمعک مشاهده گردید (P<0/05)، ولی ارتباط آماری معناداری بین آزمون اعداد دایکوتیک و میزان رضایتمندی کسب نشد (P>0/05). نتیجه‌گیری: اختلالات پردازش شنوایی مرکزی در بسیاری از بیماران دچار اختلالات شناختی مشاهده گردید. می‌توان اظهار داشت که در دوران سالمندی، علاوه بر بروز اختلال در حواس مختلف همچون شنوایی، اختلالاتی در دستگاه شناختی نیز روی می‌دهد و این می‌تواند دلیلی قوی بر نارضایتی جمع زیادی از سالمندان کم‌شنوا نسبت به سمعک و وسایل کمک شنوایی باشد.

Title: بررسی سطح استرس (تنش)، اضطراب و افسردگی سالمندان مراجعه‌کننده به کانون‌های جهان‌دیدگان شهر تهران در سال 1388

http://salmandj.uswr.ac.ir/article-1-626-fa.html

اهداف: استرس و اضطراب عوامل مهمّی در بسیاری از آسیب های روانی و اجتماعی هستند. با توجّه به آسیب‌ذیری سالمندان در برابر اثرات منفی این عوامل بر آن شدیم تا سطح استرس، اضطراب و افسردگی را در بین سالمندان مراجعه‌کننده به کانون‌های جهان‌دیدگان شهر تهران بررسی کنیم. مواد و روش‌ها: در این مطالعه توصیفی تعداد 104 سالمند، مراجعه‌کننده به کانون‌های جهان‌دیدگان شرکت کردند. پرسشنامه سنجش افسردگی، اضطراب و استرس (DASS-21) برای شرکت‌کنندگان تکمیل شد. در نهایت داده‌های جمع‌آوری‌شده با استفاده از نرم افزار SPSS 15 و آزمون های آماری، T مستقل و آنالیز واریانس یک طرفه در سطح معناداری 0/05≤P مورد تجزیه و تحلیل آماری قرار گرفتند. یافته‌ها: از 104 نفر مورد مطالعه 76 درصد زن و 24 درصد مرد به ترتیب با میانگین سنی 4/32±63/37 و 4/82±65/08 سال بودند. درصد زنان با نمره اضطراب بالا در کل از مردان بیشتر بود و این اضطراب بالا در افراد بیمار نیز بیشتر بود (P≥0/001). نتیجه‌گیری: بررسی وضعیّت افسردگی، اضطراب و استرس سالمندان در شهر تهران نشان دهنده بالا بودن این عوامل می‌باشد که اجرای طرح؟‌های مداخله‌ای برای بهبود شیوه زندگی و کاهش استرس‌ها ضروری به نظر می‌رسد.

Title: تأثیر روش درمان گروهی مرور زندگی در سالمندان مبتلا به درد مزمن

http://salmandj.uswr.ac.ir/article-1-628-fa.html

اهداف: درد مزمن یکی از مشکلات عمده در دوره سالمندی است. میزان شیوع این درد در سالمندان ایرانی مقیم خانه‌های نگهداری از سالمندان نشان می‌دهد که در هر دوره حداقل 72 درصد آنان از درد مزمن رنج می‌برند. تحقیقات نشان می‌دهند که مرور ساختار یافته زندگی در افراد سالمند می‌تواند نه تنها مفید بلکه حتی نوعی درمان محسوب شود. هدف از شیوه درمانی مرور زندگی، افزایش رضایت از زندگی و ارتقای عزت نفس است. همچنین این شیوه به فرد سالمند کمک می‌کند تا با بحران‌ها و تغییرات زندگی‌اش و آنچه ازدست داده کنار آمده و از طریق حل تعارضات گذشته، امروزش را بپذیرد. بیمار در طی درمان داستان زندگی‌اش را بازسازی کرده و با همراهی درمانگر، تجربیات مثبت و منفی را دوباره می‌آزماید. پژوهش حاضر به بررسی اثر درمان مرور زندگی بر درد مزمن سالمندان می‌پردازد. مواد و روش‌ها: 40 سالمند از 4 مرکز نگهداری از سالمندان شهر تهران انتخاب شدند. بعد از دریافت اطلاعات جمعیت شناختی، وضعیت درد آنها با کمک پرسشنامه درد مک گیل اندازه‌گیری شده و سپس به‌صورت تصادفی در دو گروه جایگزین شدند. گروه اول (20=N) تحت درمان مرور زندگی قرار گرفتند و گروه دوم (20=N) به‌عنوان گروه کنترل هیچ درمانی را دریافت نکردند. بعد از پایان دوره درمان میزان درد افراد از پرسشنامه درد مجددا اندازه‌گیری شد و با نمرات اولیه مورد مقایسه قرار گرفت. یافته‌ها: نتایج حاصل از تجزیه و تحلیل کواریانس نشان می‌دهند که بین دوگروه تفاوت معناداری وجود دارد و نمره درد افراد گروه اول پس از درمان در مقایسه با گروه کنترل کاهش یافته است. نتیجه‌گیری: با توجه به این یافته‌ می‌توان چنین نتیجه‌گیری نمود که استفاده از روش درمانی مرور زندگی، به نحو قابل توجهی دردهای مزمن دوره سالمندی را تحت تاثیر قرار داده و موجب کاهش آن می‌گردد.

Title: مقایسه عملکرد جوانان با سالمندان در انجام یک وظیفه زمان واکنش پیاپی در شرایط تکراری و تصادفی

http://salmandj.uswr.ac.ir/article-1-629-fa.html

اهداف: انجام مهارت‌های حرکتی در سالمندان اهمیت ویژه‌ای دارد. مطالعه حاضر با هدف بررسی مقایسه ای نحوه واکنش افراد به الگوهای تکراری و تصادفی در طی یک وظیفه زمان واکنش پیاپی درگروه‌های جوان و سالمند انجام شد. مواد و روش‌ها: مطالعه حاضر به شکل مقایسه‌ای تحلیلی انجام گرفت و نمونه‌ها شامل 30 جوان و 30 سالمند بود که هر گروه به دو زیرگروه 15 نفره تلویحی و صریح تقسیم شدند. جهت انجام تحقیق از یک وظیفه زمان واکنش پیاپی که با استفاده از یک نرم‌افزار کامپیوتری اعمال می‌‌شد، استفاده گردید. در طی آن 4 مربع با رنگ‌های مختلف بر صفحه نمایشگر ظاهر می‌‌شد که داوطلب به محض مشاهده هر مربع کلید تعریف شده مرتبط با آن را فشار می‌‌داد. زمان انجام بلوک‌های منظم و تصادفی بعنوان متغیر وابسته در نظر گرفته شده که با آزمون‌های t مستقل و Repeated Measures ANOVA استفاده گردید. یافته‌ها: هر دو گروه جوان در مقایسه با گروههای سالمند تمامی بلوکها را چه در الگوهای تصادفی و چه تکراری با اختلاف معنی‌داری در زمان کوتاه‌تری انجام می دادند (P<0/05). هر دو گروه سالمند نسبت به الگوی تکراری قبلی، سکانس های تصادفی را بطور معنی داری در زمان کوتاهتری انجام دادند (P<0/05) در حالیکه در گروه جوان صریح و تلویحی این تفاوت معنی دار نبود (P>0/05). گروه سالمند صریح پس از خارج شدن از الگوی تصادفی، در زمان انجام بلوک‌های 7 و 8 نسبت به بلوک 6 افزایش معنی‌داری نشان دادند (P<0/05). نتیجه‌گیری: تحقیق حاضر نشان داد که افراد جوان چه در الگوی تصادفی و چه تکراری سطح عملکرد بالاتری نسبت به افراد سالمند داشتند و افراد سالمند در انجام سکانس های تصادفی به‌طور معنی‌داری عملکرد بهتری نسبت به سکانس‌های تکراری نشان دادند.
